# Correlation between parameters at initiation of renal replacement therapy and outcome in patients with acute kidney injury

**DOI:** 10.1186/cc8154

**Published:** 2009-11-04

**Authors:** Marlies Ostermann, René WS Chang

**Affiliations:** 1 Department of Critical Care, Guy's & St Thomas' Foundation Hospital, Westminster Bridge Road, SE1 7EH, UK; 2 Department of Nephrology & Transplantation, St George's Hospital, Blackshaw Road, SW17 0QT, UK

## Abstract

**Introduction:**

Renal replacement therapy (RRT) is a fully established treatment for critically ill patients with acute kidney injury (AKI) but there are no scientifically established criteria when to initiate it. Our objectives were to describe the epidemiology of critically ill patients with AKI receiving RRT and to evaluate the relationship between biochemical, physiological and comorbid factors at time of RRT and ICU mortality.

**Methods:**

Retrospective analysis of demographic and physiologic data of 1,847 patients who received RRT for AKI in 22 ICUs in UK and Germany between 1989 - 1999.

**Results:**

54.1% of RRT patients died in ICU. ICU survivors were younger, had a lower APACHE II score and fewer failed organ systems on admission to ICU compared to non-survivors. Multivariate analysis showed that at time of initiation of RRT, independent risk factors for ICU mortality were mechanical ventilation [odds ratio (OR) 6.03], neurological failure (OR 2.48), liver failure (OR 2.44), gastrointestinal failure (OR 2.04), pre-existing chronic illnesses (OR 1.74), haematological failure (OR 1.74), respiratory failure (OR 1.62), oligoanuria (OR 1.6), age (OR 1.03), serum urea (OR 1.004) and cardiovascular failure (OR 1.3). A higher pH at initiation of RRT was independently associated with a better outcome. Failure to correct acidosis and development of more organ failure within 48 hours after initiation of RRT were also associated with an increased risk of dying in ICU.

**Conclusions:**

Oligoanuria, acidosis and concomitant dysfunction of other organs at time of RRT were associated with poor survival. In contrast, serum creatinine and urea levels only had a weak correlation with outcome after RRT.

## Introduction

Acute kidney injury (AKI) is a common problem in hospitalised patients with a reported incidence of between 10 and 20% but as high as 70% in critically ill patients in the intensive care unit (ICU) [1-4]. To date, there are no curative therapies. Management is limited to fluid and haemodynamic optimisation, and renal replacement therapy (RRT) when necessary. Furthermore, there are no robust data to accurately distinguish in advance between injured kidneys that will need extracorporeal support and kidneys that retain capacity for early recovery.

Although RRT has been an integral part of critical care for decades and technologies have advanced considerably, there are no scientifically established criteria for the initiation of RRT. As a result, the provision of renal support is very variable in clinical practice [5-7].

The indications for RRT in critically ill patients with AKI have generally been extrapolated from the end-stage kidney disease experience and included refractory hyperkalaemia, resistant fluid overload, severe persistent metabolic acidosis, and overt uraemic symptoms, including uraemic pericarditis and encephalopathy. Although there is little dispute about the necessity of RRT for these urgent indications, there is no consensus on the degree of azotaemia or the duration of AKI that warrants RRT in the absence of these 'absolute' indications [8]. Clinical studies addressing the 'optimal' timing of RRT are conflicting [9-12]. In a meta-analysis, Seabra and colleagues summarised the results of 23 studies, including four randomised controlled trials, which compared the effect of "early" versus "late" RRT on mortality in patients with AKI [12]. Despite the conclusion that early institution of RRT might have a beneficial effect on survival, the authors emphasized that the studies were very heterogenous and differed in quality. The differentiation between 'early' and 'late' RRT is variable and usually based on arbitrary thresholds in traditional parameters such as serum creatinine or urine output, time from admission to ICU or time from diagnosis of AKI [11].

In 2006, the AKI Network assembled a multidisciplinary stakeholder committee with representation from the 18 leading international professional societies of critical care and nephrology. They identified the key questions for future research in the field of AKI in ICU [13]. Top priority was given to the broad topics of epidemiology of AKI and RRT, including the quest for criteria for RRT.

The objectives of this study were twofold. Firstly, to describe the epidemiology of ICU patients treated with RRT and to compare them with AKI stage III patients who did not receive RRT. Secondly, in search of the optimal criteria for RRT, we aimed to evaluate the relation between different physiological, metabolic and comorbid factors at the time of initiation of RRT and subsequent outcome.

## Materials and methods

### Study population

Using the Riyadh Intensive Care Program database with demographic and daily physiological data of 41,972 adult patients admitted to 19 ICUs in the UK and three ICUs in Germany between June 1989 and October 1999, we analysed the data of 1847 patients who had received RRT for AKI.

Receiving RRT is one of four criteria for the diagnosis of AKI stage III according to the AKI network [14]. For comparison, we identified 935 patients with AKI stage III as defined by the creatinine criteria (ie. rise in serum creatinine to ≥ 354 μmol/L or rise in serum creatinine by >300% from baseline within 48 hours) who were not treated with RRT.

All 22 centres included in the study were able to provide RRT if necessary.

### Data analysis

Acute severity of illness was measured using the acute physiology and chronic health evaluation score (APACHE) II and Sequential Organ Failure Assessment (SOFA) scoring system. Organ system failures were determined according to the criteria by Knaus and colleagues [15].

Cardiovascular failure was defined by the presence of one or more of the following: heart rate 54 beats/min or less; mean arterial blood pressure less than 50 mmHg; occurrence of ventricular tachycardia and/or ventricular fibrillation; serum pH of 7.24 or less with a partial pressure of carbon dioxide (pCO_2_) of 49 mmHg or less.

Respiratory failure was defined as a presence of one or more of the following: respiratory rate of 5 breaths/min or less or 49 breaths/min or more; pCO_2 _50 mmHg or more; alveolar-arterial pO_2 _difference of 350 mmHg or more; dependent on ventilator on the fourth day of organ system failure (i.e. not applicable for the initial 72 hours of organ system failure).

Haematological failure was defined as the presence of one or more of the following: white blood cells of 1000 cells/mm^3 ^or less; platelets of 20,000 cells/mm^3 ^or less; haematocrit of 20% or less.

Neurological failure was defined as Glasgow Coma Score of 6 or less (in absence of sedation). If patient is sedated and/or paralysed, neurological scoring is not performed and patient is not considered in neurological failure.

Hepatic failure was defined by the presence of both: bilirubin more than 60 mg/L or a two-fold increase in serum alkaline phosphatase; prothrombin time more than four seconds over upper limit of normal range or a two-fold increase in serum aspartate aminotransferase.

We also included a definition for gastrointestinal failure, which was 'failure to tolerate enteral nutrition and need for total parenteral nutrition' [16].

The highest number of failed organs (excluding renal failure) on any day during the stay in the ICU was recorded as 'Maximum number of associated organ failure'.

We also recorded whether patients had evidence of any of the following pre-existing chronic illnesses:

Cardiovascular - New York Heart Association Class IV angina or symptoms at rest or on minimal exertion, e.g getting dressed or self-care;

Liver - Biopsy proven cirrhosis and documented portal hypertension or previous episodes of variceal bleed/liver failure/encephalopathy;

Respiratory - Chronic restrictive, obstructive, or vascular disease resulting in severe exercise restrictions, e.g. unable to climb stairs or documented chronic hypoxia, hypercapnea, secondary polycythaemia, severe pulmonary hypertension (>40 mmHg) or respiratory dependency;

Immunocompromised - Patients receiving immunosuppression, chemotherapy, radiation, long-term steroid treatment, leukaemia, lymphoma, AIDS or widespread metastatic cancer.

### Ethics approval

The local ethics committee confirmed that Research and Ethics approval was not required for this study. The need for informed consent was also waived because the study required neither an intervention nor breach of privacy or anonymity.

### Statistical analysis

Demographic data were presented as mean ± standard deviation and 95% confidence intervals (CI) or median and range. Statistical significance was evaluated in univariate analyses using chi-square test, Fisher's exact test and Mann-Whitney U test. A matrix was created to illustrate the impact of combinations of different physiological and biochemical factors at the time of RRT on subsequent outcome.

Multivariate logistic regression analyses were conducted to identify independent predictors of all-cause ICU and hospital mortality and to obtain the odds ratios (ORs). Variables that were found to be significant risk factors in univariate analyses were entered simultaneously in the multivariable model (enter method). The statistical package SPSS (Version 14.0, Woking, Surrey, UK) was used for all statistical analyses. A *P *< 0.05 was considered statistically significant.

Data related to ICU mortality are presented within this paper whereas results related to hospital mortality are available as supplementary tables in Additional data file 1 with the online version of this paper.

## Results

### Patient characteristics

For AKI, 1847 patients received RRT of whom 1473 patients (79.8%) were treated with a continuous mode alone (continuous arterio-venous haemofiltration or continuous veno-venous haemo(dia)filtration), 95 patients (5.1%) received intermittent haemodialysis (HD) and 12 patients (0.6%) had peritoneal dialysis (PD) alone. Two hundred and forty patients (13.0%) were treated with a combination of a continuous mode followed by HD. One patient had incomplete data related to the type of RRT. ICU mortality was 54.1% and hospital mortality was 61.6%. ICU survivors were younger and less sick on admission to the ICU as evidenced by a significantly lower APACHE II score and fewer failed organ systems (Table 1). In addition, they had less pre-existing chronic illnesses. During their stay in ICU, they needed ventilation less often and had fewer organ failures.

**Table 1:** Characteristics of patients on RRT

Parameter	ICU Survivors (n = 848)	ICU Non-survivors (n = 999)	***P***	OR (95% CI)
**Parameters on admission to ICU**				
Age, Mean (SD)	57.96 (17.01)	61.89 (14.19)	<0.0001	
Male sex	558 (65.9%)	684 (68.4%)	0.23	
Reason for ICU admission				
Sepsis	521 (28.2%)	316 (60.7%)		
Cardiac surgery	115 (13.6%)	121 (12.1%)		
COPD/asthma	203 (11%)	117 (57.6%)		
Cardiac disease (non-surgery)	233 (12.6%)	137 (58.8%)		
Post-abdominal surgery	109 (5.9%)	77 (70.6%)		
Neurological (non-surgical)	64 (3.5%)	13 (20.3%)		
Trauma	28 (1.5%)	16 (57.1%)		
APACHE II score	21 (1-43)	24 (3-52)	0.0001	
Median (range)				
SOFA score	10 (2-20)	11 (0-22)	0.3	
Median (range)				
Number of failed organs*	1 (0-5)	2 (0-6)	<0.0001	
Median (range)				
Emergency surgery	93 (11%)	150 (15.0%)	0.01	
Hb ≤ 9 g/dL	243 (28.7%)	253 (25.3%)	0.11	
Pre-existing chronic illness	174 (20.5%)	295 (29.5%)	<0.0001	

**Parameters during stay in ICU**				
Ventilated	710 (83.7%)	977 (97.8%)	<0.0001	8.63 (5.45-13.68)
Maximum OF in ICU	2 (0-5)	3 (0-6)	0.02	
Median (range)				

**Parameters at time of RRT**				
**Interval between ICU admission and RRT**				
< 3 days	657 (77.5%)	679 (68%)	<0.0001	
3-5 days	97 (11.4%)	159 (15.9%)	(<3 days vs ≥ 3 days)	
6-10 days	73 (8.6%)	101 (10.1%)		
>10 days	21 (2.5%)	60 (6.0%)		

**Days between biochemical diagnosis of AKI III and RRT**				
Same day or before	1,568 (84.9%)	832 (53.1%)		
1-2 days later	67 (3.6%)	44 (65.7%)		
3-5 days later	37 (2.0%)	24 (64.9%)		
6-10 days later	69 (3.7%)	32 (46.4%)		
>10 days later	106 (5.7%)	57 (53.8%)		
				
**Biochemistry**, Mean (SD)				
Serum urea (mmol/L) (excl 31 pts)	24.1 (13.6)	24.23 (13.21)	0.84	
Serum creatinine (umol/L)(excl 50 pts)	370.9 (269.9)	312 (177.9)	<0.0001	
pH (excl 29 pts)	7.34 (0.13)	7.27 (0.16)	<0.0001	
Serum HCO_3 _(umol/L) (excl 572 pts)	22.1 (5.94)	20.05 (6.24)	0.11	
Serum K (mmol/L) (excl 30 pts)	4.55 (1.11)	4.63 (1.06)	0.17	
				
**Organ failure**				
CVS failure	243 (28.7%)	446 (44.6%)	<0.0001	2.01 (1.65-2.44)
RS failure	349 (41.2%)	615 (61.6%)	<0.0001	2.29 (1.90-2.76)
GI failure	129 (15.2%)	299 (29.9%)	<0.0001	2.38 (1.89-3.00)
NEURO failure	50 (5.9%)	160 (16.0%)	<0.0001	3.04 (2.18-4.24)
HAEM failure	62 (7.2%)	116 (11.6%)	0.002	1.67 (1.21-2.30)
HEP failure	24 (2.8%)	80 (8.0%)	<0.0001	2.99 (1.88-4.76)
CVS + RS failure	92 (10.9%)	260 (26.0%)	<0.0001	2.89 (2.23-3.74)
				
Total OF (excluding renal failure)				
Median (range)	2 (0-5)	2 (0-6)	<0.0001	
MAP (mm Hg) (excl 29 pts)	76.6 (26.1)	60.5 (21.01)	<0.0001	
Oligoanuria (urine ≤ 400 ml/hr)	402 (47.3%)	597 (59.8%)	<0.0001	1.65 (1.37-1.98)
Median SOFA score [range]	10 [0-21]	12 [0-21]	<0.0001	

**Type of RRT**				
CRRT alone	665 (78.4%)	808 (80.9%)	0.2	1.16 (0.93-1.46)
IHD alone	54 (6.4%)	41 (4.1%)	0.03	0.63 (0.41-0.95)
PD alone	7 (0.8%)	5 (0.5%)	0.4	0.60 (0.19-1.91)
CRRT + IHD	116 (13.7%)	124 (12.4%)	0.45	0.89 (0.68-1.17)
CRRT + PD	5 (0.6%)	7 (0.7%)	1	1.19 (0.38-3.76)

* excluding renal failure. AKI = acute kidney injury; APACHE = acute physiology and chronic health evaluation score; CI = confidence interval; COPD = chronic obstructive pulmonary disease; CRRT = continuous renal replacement therapy; CVS = cardiovascular; excl = excluding; GI = gastrointestinal; HAEM = haematological; Hb = haemoglobin; HEP = liver; ICU = intensive care unit; IHD = intermittent haemodialysis; MAP = mean arterial blood pressure; NEURO = neurological; OF = organ failure; OR = odds ratio; PD = peritoneal dialysis; pts = patients; RRT = renal replacement therapy; RS = respiratory; SD = standard deviation; SOFA score = Sequential Organ Failure Assessment score.

RRT is one of five criteria for the diagnosis of AKI stage III and 935 patients in this study fulfilled the creatinine criteria for AKI stage III but were not treated with RRT. Direct comparison confirmed that patients with AKI stage III on RRT had a significantly higher ICU and hospital mortality than patients with AKI III not treated with RRT (Table 2). However, patients on RRT were sicker as evidenced by higher APACHE II and SOFA scores on admission to the ICU, more failed organ systems whilst in ICU and a higher need for mechanical ventilation.

**Table 2:** Comparison between AKI III on RRT and AKI III without RRT

Factor	AKI III on RRT (n = 1847)	AKI III without RRT (n = 935)	*P*
**Male sex**	1242 (67.2%)	660 (70.6%)	0.08
**Age**	60.09 (15.68)	63.14 (15.42)	0.28
Mean (SD)			
**APACHE II on admission to ICU**	22 (1-52)	19 (2-46)	<0.0001
Median (range)	22.92 (7.80)	19.84 (6.63)	
Mean (SD)			
**SOFA score on admission to ICU**	10 (0-22)	7 (0-17)	0.0063
Median (range)	10.23 (3.18)	7.61 (2.96)	
Mean (SD)			
Organ failure on admission to ICU*	2 (0-6)	1 (0-4)	<0.0001
Median (range)	1.72 (1.20)	1.11 (0.95)	
Mean (SD)			
Maximum organ failure during ICU*	2 (0-6)	2 (0-6)	
Median (range)	2.27 (1.17)	1.67 (0.97)	<0.0001
Mean (SD)			
**Mechanical ventilation**	1,687 (91.3%)	729 (77.97%)	<0.0001
**Pre-existing chronic illness**	469 (25.4%)	238 (25.5%)	0.97
**Hb ≤ 9 g/dl on admission to ICU**	496 (26.9%)	193 (20.6%)	0.0004
**Cardiac surgery**	236 (12.8%)	95 (10.2%)	0.051
**Mortality**			
ICU mortality	999 (54.1%)	380 (40.6%)	<0.0001
Hospital mortality	1138 (61.6%)	472 (50.5%)	<0.0001

* excluding acute kidney injury. AKI = acute kidney injury; APACHE = acute physiology and chronic health evaluation score; Hb = haemoglobin; ICU = intensive care unit; RRT = renal replacement therapy; SD = standard deviation; SOFA score = Sequential Organ Failure Assessment score.

Only 573 of all 1847 patients who received RRT, fulfilled the creatinine criteria for AKI stage III. Of the remaining patients, 691 were oliguric with a urine output less than 400 ml/day and would have (probably) fulfilled the urine criteria for AKI stage III. The remaining 583 patients had RRT without a 300% change in creatinine or a creatinine of 354 μmol/L or more (as per AKI stage III classification) or without being oliguric.

### Parameters at time of initiation of RRT

Among patients treated with RRT, ICU survivors were characterised by a significantly higher mean serum pH, higher mean arterial blood pressure, fewer failed organ systems and a higher serum creatinine value at time of initiation of RRT compared with non-survivors (Table 1). There was no significant difference in mean serum urea, HCO_3 _and K^+ ^levels between ICU survivors and non-survivors. Respiratory failure was the most common associated organ failure at the time of RRT (52.2%), followed by cardiovascular failure in 37.3% of patients. Only 5.6% of patients had hepatic failure when RRT was started but ICU mortality in this group was high at 76.9%.

Of all survivors, 6.4% had intermittent haemodialysis compared with only 4.1% of the non-survivors. There was no other significant difference in the types of RRT between survivors and non-survivors.

The majority of patients (72.3%) were initiated on RRT within the first two days after admission to ICU. Of these patients, 49.2% survived to discharge from ICU. In contrast, among patients who started RRT six days or longer after admission to ICU only 36.9% survived (Table 1).

Using serum urea of 27.1 mmol/L (76 mg/dL) as a cut-off level between early and late RRT (as suggested by Liu and colleagues [9]), we found no significant difference in ICU and hospital outcome between both groups (Table 3 and Table S1 in Additional data file 1). Similarly, a serum urea cut-off of 24.2 mmol/L (as per Bagshaw and colleagues [11]) did not differentiate between survivors and non-survivors either.

**Table 3:** Parameters at initiation of RRT and ICU outcomeay

Variables on day of RRT	Incidence (n = 1847)	ICU mortality	*P*	OR (95% CI)
**Serum creatinine (μmol/L)**				
≤ 200	456 (24.7%)	255 (55.9%)	(≤ 500 vs >500)	
>200-500	1064 (57.6%)	609 (57.2%)	<0.0001	
>500	278 (15.1%)	102 (36.7%)		2.27 (1.75-2.96)
≤ 309	953 (51.6%)	564 (59.2%)	<0.0001	
>309 (50 data missing)	848 (45.9%)	404 (47.6%)		1.59 (1.32-1.92)
**Serum pH**				
<7.2	397 (21.5%)	294 (74.1%)	(<7.2 vs	
7.2-7.35	675 (36.5%)	385 (57.0%)	≥ 7.2) <0.0001	
>7.35 (28 data missing)	747 (40.4%)	299 (40.0%)		0.32 (0.25-0.42)
**Serum K^+ ^(mmol/L)**				
≤ 6	1634 (88.5%)	877 (53.7%)		
>6 (28 data missing)	185 (10.0%)	101 (54.6%)	0.82	0.96 (0.71-1.31)
**Urine output**				
<400 ml/24 hours	994 (53.8%)	595 (59.9%)		
≥ 400 ml/24 hours	853 (46.2%)	404 (47.4%)	<0.0001	1.66 (1.38-1.99)
**Serum HCO_3 _(mmol/L)**				
<10	50 (2.7%)	37 (74%)	(<15 vs	
10-14.9	146 (7.9%)	103 (70.5%)	≥ 15)	
≥ 15 (577 data missing)	1074 (58.1%)	565 (52.6%)	<0.0001	2.25 (1.62-3.14)
**Serum urea (mg/dL)**				
≤ 27.1 mmol/L	1186 (64.2%)	641 (54.0%)	1	
>27.1 mmol/L	661 (35.8%)	358 (54.2%)		
(30 data missing)				
**MAP (mmHg)**				
≤ 65	1,116 (60.4%)	718 (64.3%)		
>65 (28 data missing)	703 (38.1%)	260 (37.0%)	<0.0001	3.07 (2.53-3.74)
**SOFA CVS score**				
**0**	270 (14.6%)	73 (27%)	SOFA ≤ 2	
**1**	206 (11.2%)	77 (37.4%)	versus	
**2**	494 (26.7%)	253 (51.2%)	SOFA >2	
**3**	0	-		
**4**	877 (47.5%)	596 (67.96%)	<0.0001	2.98 (2.47-3.61)
**SOFA RESP score**				
**0**	21 (1.1%)	17 (80.95%)		
**1**	0	-		
**2**	225 (12.2%)	43 (19.1%)		
**3**	0	-		
**4**	1601 (86%)	939 (58.7%)	<0.0001	4.4 (3.23-5.98)
**SOFA NEURO score**				
**0**	1352 (73.2%)	686 (50.7%)		
**1**	68 (3.7%)	30 (44.1%)		
**2**	109 (5.9%)	59 (54.1%)		
**3**	145 (7.9%)	88 (60.7%)	<0.0001	2.32 (1.79-3.01)
**4**	173 (9.4%)	136 (78.6%)		
**SOFA COAG score**				
**0**	739 (40%)	368 (49.8%)		
**1**	597 (32.3%)	331 (55.4%)		
**2**	511 (27.7%)	300 (58.7%)		
**3**	0	-		
**4**	0	-	N/A	
**SOFA LIVER score**				
**0**	1125 (60.9%)	550 (48.9%)		
**1**	206 (11.2%)	129 (62.6%)		
**2**	140 (7.6%)	292 (15.8%)		
**3**	188 (64.4%)	84 (60.0%)		
**4**	84 (4.5%)	48 (57.1%)	0.133	1.25 (0.94-1.66)
**Total SOFA score**				
≤ 12	1149 (62.2%)	517 (45.0%)		
>12 (21 data missing)	677 (36.7%)	465 (68.7%)	<0.0001	0.37 (0.31-0.46)
**Organ failure**				
CVS failure	689 (37.3%)	446 (64.7%)		
No CVS failure	1,158 (62.7%)	553 (47.8%)	<0.0001	2.01 (1.65-2.44)
RS failure	964 (52.2%)	615 (63.8%)		
No RS failure	883 (47.8%)	384 (43.5%)	<0.0001	2.29 (1.90-2.76)
GI failure	428 (23.2%)	299 (69.9%)	<0.0001	2.38 (1.89-3.00)
No GI failure	1419 (76.8%)	700 (49.3%)		
NEURO failure	210 (11.4%)	160 (76.2%)	<0.0001	3.04 (2.18-4.24)
No NEURO failure	1,637 (88.6%)	839 (51.3%)		
HAEM failure	180 (9.7%)	118 (65.6%)		
No HAEM failure	1,667 (90.3%)	881 (52.8%)	0.0012	1.70 (1.23-2.34)
HEP failure	104 (5.6%)	80 (76.9%)		
No HEP failure	1743 (94.4%)	919 (52.7%)	<0.0001	2.99 (1.88-4.76)
CVS + RS failure	352 (19.1%)	260 (73.9%)		
No CVS + no RS failure	546 (29.6%)	198 (36.3%)	<0.0001	4.97 (3.70-6.67)
**Total number of failed organs**				
0	129 (6.98%)	34 (26.4%)		
1	437 (23.7%)	166 (38.0%)		
2	683 (37.0%)	365 (53.4%)		
3	389 (21.1%)	255 (65.6%)		
>3	209 (11.3%)	179 (85.6%)		

CI = confidence interval; COAG = coagulation; CVS = cardiovascular; excl = excluding; GI = gastrointestinal; HAEM = haematological; HEP = liver; ICU = intensive care unit; MAP = mean arterial blood pressure; NEURO = neurological; OR = odds ratio; pts = patients; RRT = renal replacement therapy; RESP/RS = respiratory; SOFA score = Sequential Organ Failure Assessment score; vs = versus.

Univariate analysis showed that at the time of initiation of RRT, the following factors were associated with an increased risk of dying: low serum pH, oligoanuria, low serum (HCO_3_), total SOFA score above 12, mean arterial blood pressure of 65 mmHg or less, cardiovascular SOFA score above 2, respiratory SOFA score above 2, neurological SOFA score above 2, different types of organ failures and a lower serum creatinine level (Table 3 and Table S1 in Additional data file 1). The presence of more than one risk factor increased the risk of death further. Different combinations of risk factors were associated with different mortality rates (Table 4 and Table S2 in Additional data file 1).

**Table 4:** Combinations of parameters at time of RRT and ICU mortality

Parameter at time of RRT	Serum pH <7.2	Urine <400 ml/24 hours	SOFA score >12	MAP ≤ 65	CVS failure	RESP failure	HEP failure
**Serum pH <7.2**	74.10%						
**Urine <400 ml/24 hours**	79.10%	59.90%					
**SOFA score >12**	84.50%	72.80%	68.70%				
**MAP ≤ 65**	77.30%	69.50%	76.50%	64.60%			
**CVS failure**	75.20%	70.40%	76%	71%	64.70%		
**RESP failure**	79%	70.50%	75%	72.30%	73.90%	63.80%	
**HEP failure**	92%	77%	80.90%	85.30%	88.70%	83.90%	76.90%
Total OF 1 *	61.10%	37.40%	52%	50.70%	-	-	-
Total OF 2 *	69.90%	56.60%	60%	60.30%			
Total OF 3 *	74.40%	69.50%	73.30%	72%			
Total OF >3 *	89.70%	89.10%	89.20%	88.80%			
**Days since ICU admission**							
**<3**	71.30%	57.10%	67.70%	61.30%	63.40%	62.60%	75.60%
**5-Mar**	94.70%	69.70%	71.80%	74%	71.40%	65.20%	76.90%
**10-Jun**	83.30%	64.80%	65.70%	69.10%	65.90%	59.70%	75%
**>10**	84.60%	81.80%	78.60%	85.40%	76.90%	76.70%	100%

* excluding acute kidney injury. CVS = cardiovascular; HEP = liver; ICU = intensive care unit; MAP = mean arterial blood pressure in mmHg; OF = failed organ system(s); RESP = respiratory; RRT = renal replacement therapy; SOFA = Sequential Organ Failure Assessment.

### Timing of RRT in relation to onset of AKI III

Patients who received RRT before they met the creatinine criteria for AKI stage III (serum creatinine ≥ 354 μmol/L (≥ 4.0 mg/dL) or a rise in serum creatinine by more than 300% from baseline) had a significantly lower ICU mortality than patients who were started on RRT on the day when they met the AKI stage III criteria (49.8% versus 64.6%; *P *< 0.0001). This group also had a better ICU outcome compared with patients in whom RRT was initiated after the AKI criteria were fulfilled (49.8% versus 56.3%) but this difference did not reach statistical significance (*P *= 0.05).

### Multivariate analysis

In a multivariate analysis, mechanical ventilation and associated neurological failure on the day of RRT were the strongest independent risk factors for ICU and hospital mortality, followed by liver and gastrointestinal failure, haematological failure and pre-existing chronic health problems (Table 5 and Table S3 in Additional data file 1). A higher serum pH was independently associated with a better outcome. Serum creatinine and urea concentrations on the day of RRT exhibited opposite associations: a raised urea and a low creatinine concentration were independent risk factors for dying. Gender, non-surgical admission, emergency surgery and cardiac surgery were not independently associated with ICU and hospital mortality.

**Table 5:** Multivariate analysis: parameters on day of RRT affecting ICU outcome of patients with AKI treated with RRT

Parameter	B	SE	*P*	OR	95% CI
**PH on day of RRT**	-3.719	0.448	<0.0001	.024	0.01-0.06
**(creatinine) on day of RRT**	-0.002	0.000	<0.0001	.998	0.997-0.998
**(urea) on day of RRT**	0.004	0.001	<0.0001	1.004	1.002-1.005
**Age**	0.026	0.004	<0.0001	1.026	1.02-1.03
**CVS failure on day of RRT**	0.261	0.127	0.039	1.299	1.01-1.66
**Oligoanuria on day of RRT**	0.469	0.115	<0.0001	1.599	1.28-2.00
**RESP failure on day of RRT**	0.485	0.114	<0.0001	1.624	1.299-2.03
**HAEM failure on day of RRT**	0.553	0.207	0.008	1.738	1.16-2.61
**Pre-existing chronic illness**	0.554	0.129	<0.0001	1.740	1.35-2.24
**GI failure on day of RRT**	0.714	0.135	<0.0001	2.042	1.57-2.66
**HEP failure on day of RRT**	0.893	0.273	0.001	2.441	1.43-4.17
**NEURO failure on day of RRT**	0.908	0.197	<0.0001	2.479	1.69-3.64
**Ventilated**	1.797	0.270	<0.0001	6.029	3.56-10.23
**male gender**	0.153	0.120	0.200	1.166	0.92-1.47
**post non-surgical admission**	0.217	0.191	0.255	1.243	0.86-1.81
**post cardiac surgery**	-0.062	0.211	0.770	.940	0.62-1.42
**post-emergency surgery**	0.399	0.212	0.060	1.491	0.98-2.26
**Constant**	26.653	3.279	<0.0001	37587255 4286.662	

Exclusion of patients with missing data for creatinine, urea, MAP, pH; patients included n = 1794. The area under the receiver operator characteristic curve was 0.784 (Hosmer-Lemeshow chi-squares = 7.769; two degrees of freedom, *P *= 0.456).

AKI = acute kidney injury; CI = confidence interval; CVS = cardiovascular; GI = gastrointestinal; HAEM = haematological; HEP = hepatic; ICU = intensive care unit; NEURO = neurological; OR = odds ratio; RESP = respiratory; RRT = renal replacement therapy; SE = standard error.

### Progress after initiation of RRT

Due to the dynamic nature of critical illness, prognosis of patients on RRT not only depends on severity of illness at time of initiation of RRT but also on subsequent progress. Patients who developed more organ failure in the 48-hour period after starting RRT had a significantly higher ICU mortality than patients without any deterioration, ie. patients who still had the same number of or fewer failed organ systems (Table 6). The same trend was seen when comparing the serum pH at initiation of RRT with the pH on the following day. Patients whose pH fell had a significantly higher ICU mortality than those in whom serum pH remained unchanged or rose.

**Table 6:** Impact of changes after initiation of RRT on ICU outcome

Response after initiation of RRT	Incidence	ICU mortality	*P* (worsened versus unchanged)	*P* (worsened versus improved)
**Number of failed organs 48 hours later**:				
**unchanged**	583 (31.6%)	304 (52.1%)		
**increased by 1**	272 (14.7%)	191 (70.2%)	<0.0001	<0.0001
**increased by ≥ 2**	319 (17.3%)	301 (94.4%)		
**reduced**	673 (36.4%)	203 (30.2%)		
				
**Serum pH 24 hours later**:				
**unchanged**	172 (9.3%)	86 (50%)	0.061	0.008
**reduced**	755 (40.9%)	439 (58.1%)		
**increased**	920 (49.8%)	474 (51.5%)		

ICU = intensive care unit; RRT = renal replacement therapy.

One hundred and thirty-nine RRT patients survived their stay in the ICU but died later in hospital. The exact causes of death are not available. However, 42 patients (30.2%) had pre-existing chronic illnesses. Furthermore, at time of discharge from the ICU, 65 patients (46.7%) had persistent severe AKI as evidenced by a serum urea of 36 mmol/L or more, serum creatinine of 310 μmol/L or more or ongoing need for RRT.

## Discussion

This study confirms that timing of RRT in critically ill patients with AKI remains a very complex process. Risk factors for death in the ICU were a low serum pH, oligoanuria, mechanical ventilation and associated organ failure at time when RRT was initiated. Failure to correct acidosis or development of new organ failure within 48 hours after RRT was started, were additional markers of a poor outcome.

Despite the fact that RRT has become an integral part of modern critical care, identifying the optimal timing and optimal indication for RRT remains a difficult problem in clinical practice [17]. The literature contains several studies, which show a survival benefit with early initiation of RRT [9,11]. However, as documented by Bagshaw and colleagues, the differentiation between 'early' and 'late' RRT is variable and usually based on arbitrary thresholds in traditional parameters such as serum urea, serum creatinine, urine output, time from ICU admission or time from diagnosis of AKI [11]. Most evidence in favour of 'early' RRT stems from retrospective observational studies. These studies are open to the criticism that improved outcomes with 'early' RRT may simply reflect inclusion of patients with a lesser degree of organ injury and better prognosis regardless of treatment strategies. Secondly, the validity of retrospective observational data may be limited by the exclusion of patients with AKI who met criteria for early initiation of RRT but never received it. Finally, in clinical practice, it is likely that 'earlier' initiation of RRT is prompted by volume overload and/or severe electrolyte and metabolic disturbances, whereas 'late' initiation of renal support is triggered by progressive uraemia. Whether there is a relation between these different indications and outcome is not known. The literature contains four randomized controlled trials in medical patients, post-cardiac surgery and in patients with severe sepsis [18-21] of which one is only available in abstract form [21]. Although three studies showed a survival benefit with early initiation, the exact criteria for 'early' differed in all studies. Thus, current data in the literature remain inadequate to answer the question related to optimal timing of RRT. If early initiation of RRT is indeed advantageous, it remains unclear whether this benefit may be attributable to earlier clearance of uraemic toxins, earlier metabolic/uraemic control, prevention and management of volume overload, attenuation of the inflammatory process or any other effects not yet elucidated. The counterargument to a strategy of early initiation of RRT is that patients who would recover renal function with conservative treatment alone may be subjected to unnecessary risks.

In the absence of clear data whether early RRT is better than late RRT, it may be more useful to focus on identifying the optimal triggers when to start and to provide guidance towards a more individualised approach to patients with severe AKI. In our patient population, ICU survival was significantly better in patients who commenced RRT before they fulfilled the creatinine criteria for AKI stage III compared with those who started RRT on the same day as the criteria were fulfilled. We also showed that marked acidosis and oligoanuria at time of RRT were associated with an increased risk of dying. In contrast, patients who were initiated on RRT with a serum creatinine less than 500 μmol/L had a higher ICU mortality than those with a higher value. Previous studies have demonstrated similar results where both low creatinine and high urea values at the start of RRT were associated with a worse outcome in AKI patients [11,22,23]. Bagshaw and colleagues analysed the data of 1260 ICU patients treated with RRT and found that the median serum creatinine at time of RRT was 309 μmol/L [11]. Crude hospital mortality was 71.4% in patients who had a serum creatinine of 309 μmol/L or less when RRT was initiated compared with 53.4% in patients with a higher value.

Although the exact reasons for this observation are not clear, there are several plausible explanations. First, a low serum creatinine concentration may be a reflection of low muscle mass and therefore a surrogate marker of overall health status. Second, serum creatinine may also be low as a result of associated volume overload, a condition which has been found to be independently associated with mortality in ICU patients. Third, patients who start RRT with a lower serum creatinine may indeed start RRT for reasons other than azotaemia (ie. severe acidosis, fluid overload). It is possible that different indications for RRT are associated with different outcomes.

The role of serum urea as a trigger for RRT is also controversial, especially as urea is dependent on many non-renal factors [9,22]. Liu and colleagues analysed the data of 243 patients on RRT and found a significantly higher risk of death at 60 days when serum urea level was 27.1 mmol/L or more (≥ 76 mg/dL) at time of RRT [9]. Adjusted for age, hepatic failure, sepsis, thrombocytopenia and serum creatinine, the relative risk of death associated with initiation of RRT at a higher urea level was 1.85 (95% CI 1.16 to 2.96). In contrast, in our significantly larger patient population, we did not find any difference when using this cut-off. Instead, in a multivariate analysis, the most important independent prognostic factors among patients on RRT were mechanical ventilation, any associated organ failure, pre-existing chronic health problems, oligoanuria and a low serum pH at time of RRT.

Based on our data, we conclude that the decision when to initiate RRT should depend less on serum creatinine or urea levels but more on degree of acidosis, urine output and associated organ failure. The most likely answer to the search for the optimal timing of RRT is that sick AKI patients with significant metabolic disturbances and associated organ dysfunction benefit from 'earlier' initiation, whereas RRT can be delayed in less sick patients without causing harm. The key will be to identify the optimal triggers for RRT and optimal indications for RRT to enable a more individualised approach towards patients with severe AKI. Several factors beyond urea and creatinine are important when deciding whether renal support would be beneficial which calls for a more individualised approach.

Our data also confirm that outcome not only depends on the circumstances at time of RRT but also on events occurring in the subsequent days. Patients whose serum pH decreased or who developed more organ failure within 24 to 48 hours after starting RRT, had a significantly higher ICU mortality than patients whose pH improved or who had fewer failed organ systems. Maccariello and colleagues previously described the outcomes of 260 ICU patients with AKI in response to changes in associated organ dysfunction during the first three days of RRT and made similar conclusions [24]. Organ dysfunction was determined by the SOFA score (excluding renal points) on the first and third day of RRT. Patients in whom SOFA scores worsened or remained unchanged, had a poorer hospital mortality than patients in whom SOFA scores improved (80% versus 84% versus 61%; *P *= 0.003). These results clearly illustrate that the outcome of critically ill patients on RRT does not depend on single parameters but more on combinations of various factors and physiological responses during the dynamic course of the critical illness.

There are several limitations to our study. First, as a retrospective observational study it is potentially prone to bias. Second, our database does not include any data on the exact indications for RRT, including pulmonary oedema or fluid overload. We also did not have any data on the reasons why RRT was not offered to 935 patients with AKI stage III. Third, we did not record the dose of RRT. However, recent studies showed that the correlation between dose of RRT and outcome is less clear [7,25,26]. In addition, we did not record any data related to daily fluid balance and are unable to comment on the impact of fluid overload. We also did not have any data prior to ICU admission, including baseline renal function. Although we excluded patients with end-stage renal failure on long-term dialysis, we do not know how many patients had pre-existing chronic kidney disease. We accept that patients with CKD may have had a higher risk of developing acute-on-chronic renal failure and needing RRT compared with patients with normal baseline renal function. We also did not have any data on causes of death. Lastly, our database includes data from a 10-year period until 1999. It is possible that the practice of RRT has changed with advances in clinical practice. Future studies, ideally prospective randomized controlled trials will have to determine whether RRT triggered by single or multiple parameters like pH less than 7.2 and/or urine output less than 400 ml/24 hours indeed leads to a better outcome. With the discovery of new AKI biomarkers, it may also be possible to identify new, yet unknown triggers for RRT.

## Conclusions

Using a large database of more than 40,000 patients, we showed that 4.4% of all patients admitted to ICU needed RRT for AKI. In these patients, at time of initiation of RRT, the most important independent risk factors for ICU and hospital mortality were mechanical ventilation, associated organ failure, pre-existing chronic end-stage diseases and oligoanuria. A higher serum pH was independently associated with a better outcome whereas serum creatinine and urea values correlated poorly with outcome. Based on these data, the decision when to start RRT should be guided more by serum pH, urine output and associated comorbid factors rather than categorical creatinine and urea values.

## Key messages

• The practice of RRT in critically ill patients with acute kidney injury remains variable with no agreed consensus on the optimal criteria for starting.

• In patients receiving renal support, failure of other organ systems, oligo-anuria and acidosis at time of initiation of RRT and pre-existing chronic illnesses were independent risk factors for ICU and hospital mortality.

• Failure to correct acidosis and development of more organ failure within 48 hours after initiation of RRT were associated with increased ICU mortality.

• The decision when to start RRT for acute kidney injury should be guided more by associated dysfunction of other organ systems, urine output and serum pH rather than absolute serum creatinine and/or urea levels.

## Abbreviations

AKI: acute kidney injury; APACHE: acute physiology and chronic health evaluation score; CI: confidence interval; HD: intermittent haemodialysis; ICU: intensive care unit; OR: odds ratio; pCO2: partial pressure of carbon dioxide; PD: peritoneal dialysis; RRT: renal replacement therapy; SOFA: Sequential Organ Failure Assessment.

## Competing interests

The authors declare that they have no competing interests.

## Authors' contributions

RC organised the data collection to build the database using the Riyadh ICU Program, whose main strength is daily data collection until discharge or death, tracking the dynamic changes in the ICU. Both authors extracted the data from the database and performed the analyses. MO wrote the draft and RC provided critiques. Both authors approved the final manuscript.

## Supplementary Material

Additional file 1 Word file containing a table that lists the parameters at initiation of renal replacement therapy (RRT) and hospital outcome (Table S1 in Additional data file 1), a table that lists the correlation between different combinations of parameters at time of RRT and hospital mortality (Table S2 in Additional data file 1), and an additional table showing the results of a multivariate analysis of parameters on the day of RRT and the associated independent risk of hospital mortality (Table S3 in Additional data file 1). Click here for file
