# Supplementary material for: Correlation between parameters at initiation of renal replacement therapy and outcome in patients with acute kidney injury
Source: Crit Care. 2009 Nov 4;13(6):R175. doi: 10.1186/cc8154 (PMC2811955; doi:10.1186/cc8154)
Supplement: Additional file 1 — Word file containing a table that lists the parameters at initiation of renal replacement therapy (RRT) and hospital outcome (Table S1 in Additional data file 1), a table that lists the correlation between different combinations of parameters at time of RRT and hospital mortality (Table S2 in Additional data file 1), and an additional table showing the results of a multivariate analysis of parameters on the day of RRT and the associated independent risk of hospital mortality (Table S3 in Additional data file 1). [file cc8154-S1.DOC]

**Table S1 Parameters at initiation of RRT** **and hospital outcome**

| **Variables**  **on day of RRT** | **Incidence**  (n=1,847) | **Hospital**  **mortality** | **p** | **OR**  **(95% CI)** |
| --- | --- | --- | --- | --- |
| **Serum Creatinine (μmol/L)**  ≤ 200  > 200 – 500  > 500  ≤ 309  >309 (50 data missing) | 456 (24.7%)  1,064 (57.6%)  278 (15.1%)  953 (51.6%)  848 (45.9%) | 288 (63.2%)  681 (64%)  131 (47.1%)  622 (65.3%)  478 (56.4%) | <0.0001  (≤500 vs >500)  <0.0001 | 1.97 (1.53-2.55)  1.46 (1.20-1.76) |
| **Serum pH**  < 7.2  7.2 – 7.35  > 7.35 (28 data missing) | 397 (21.5%)  675 (36.5%)  747 (40.4%) | 317 (79.8%)  433 (64.1%)  367 (49.1%) | <0.0001  (pH <7.2  vs pH≥ 7.2) | 3.08 (2.36-4.02) |
| **Serum K^+^ (mmol/L)**  ≤ 6  > 6 (28 data missing) | 1,634 (88.5%)  185 (10.0%) | 997 (61%)  120 (64.9%) | 0.34 | 0.85 (0.62-1.17) |
| **Urine output**  < 400 ml/24 hours  ≥ 400 ml/24 hours | 994 (53.8%)  853 (46.2%) | 685 (68.9%)  453 (53.1%) | <0.0001 | 1.96 (1.62-2.37) |
| **Serum HCO_3_ (mmol/L)**  < 10  10 – 14.9  ≥ 15 (577 data missing) | 50 (2.7%)  146 (7.9%)  1,074 (58.1%) | 40 (80%)  114 (78.1%)  643 (59.9%) | <0.0001  (HCO_3_ <15  vs HCO_3_ ≥15) | 2.46 (1.71-3.53) |
| **Serum Urea (mg/dL)**  ≤27.1 mmol/L  >27.1 mmol/L  (30 data missing) | 1,186 (64.2%)  661 (35.8%) | 709 (59.8%)  405 (61.3%) | 0.55 | 0.94 (0.77-1.14) |
| **MAP (mm Hg)**  ≤ 65  > 65 (28 data missing) | 1,116 (60.4%)  703 (38.1%) | 799 (71.6%)  318 (45.2%) | <0.0001 | 3.05 (2.51-3.72) |
| **SOFA CVS score**  **0**  **1**  **2**  **3**  **4**  **SOFA RESP score**  **0**  **1**  **2**  **3**  **4**  **SOFA NEURO score**  **0**  **1**  **2**  **3**  **4** | 270 (14.6%)  206 (11.2%)  494 (26.7%)  0  877 (47.5%)  21 (1.1%)  0  225 (12.2%)  0  1,601 (86%)  1,352 (73.2%)  68 (3.7%)  109 (5.9%)  145 (7.9%)  173 (9.4%) | 100 (37%)  115 (55.8%)  288 (58.3%)  -  635 (72.4%)  17 (81%)  -  81 (36%)  -  1,040 (65%)  796 (58.9%)  38 (55.9%)  69 (63.3%)  93 (64.1%)  142 (82.1%) | Sofa ≤2 vs Sofa >2  <0.0001  <0.0001  <0.0001 | - 1. (2.01-2.96)   2. (2.13-3.69)   1.96 (1.50-2.57) |

**Continuation of Table S1**

| **Variables**  **on day of RRT** | **Incidence**  (n=1,847) | **Hospital**  **mortality** | **p** | **OR**  **(95% CI)** |
| --- | --- | --- | --- | --- |
| **SOFA COAG score**  **0**  **1**  **2**  **3**  **4**  **SOFA LIVER score**  **0**  **1**  **2**  **3**  **4** | 739 (40%)  597 (32.3%)  511 (27.7%)  0  0  1,125 (60.9%)  206 (11.2%)  292 (15.8%)  140 (7.6%)  84 (4.5%) | 433 (58.6%)  378 (63.3%)  327 (64%)  -  -  654 (58.1%)  142 (68.9%)  199 (68.2%)  92 (65.7%)  51 (60.7%) | -  0.51 | 1.11 (0.83-1.49) |
| **Total SOFA score**  ≤ 12  > 12 (21 data missing) | 1,149 (62.2%)  677 (36.7%) | 632 (55%)  489 (72.2%) | <0.0001 | 2.13 (1.73-2.61) |
| **Organ failure**  CVS failure  No CVS failure  RS failure  No RS failure  GI failure  No GI failure  NEURO failure  No NEURO failure  HAEM failure  No HAEM failure  HEP failure  No HEP failure  CVS + RS failure  No CVS + no RS failure | 689 (37.3%)  1,158 (62.7%)  964 (52.2%)  883 (47.8%)  428 (23.2%)  1,419 (76.8%)  210 (11.4%)  1,637 (88.6%)  180 (9.7%)  1,667 (90.3%)  104 (5.6%)  1,743 (94.4%)  352 (19.1%)  546 (29.6%) | 484 (70.2%)  654 (56.5%)  670 (69.5%)  468 (53%)  317 (74.1%)  821 (57.9%)  166 (79%)  972 (59.4%)  134 (74.4%)  1,004 (60.2%)  84 (80.8%)  1,054 (60.5%)  269 (76.4%)  253 (46.3%) | <0.0001  <0.0001  <0.0001  <0.0001  <0.0001  <0.0001  <0.0001 | 1.82 (1.49-2.22)  2.02 (1.67-2.45)   - 1. (1.64-2.65)   2. (1.83-3.65)   1.92 (1.36-2.73)   - 1. (1.67-4.51)   3.75 (2.79-5.06) |
| **Total number of OF**  0  1  2  3  > 3 | 129 (6.98%)  437 (23.7%)  683 (37.0%)  389 (21.1%)  209 (11.3%) | 42 (32.6%)  212 (48.5%)  422 (61.8%)  280 (72%)  182 (87.1%) |  |  |

*Abbreviations: OR = odds ratio; ICU = intensive care unit; OF = failed organ system(s); RRT = renal replacement therapy; SOFA = Sequential Organ Failure Assessment; CVS = cardiovascular; RS/RESP = respiratory; MAP = mean arterial blood pressure in mmHg; HEP = liver; GI = gastrointestinal; NEURO = neurological; HAEM= haematological; COAG = coagulation; vs = versus; excl = excluding; pts = patients*

**Table S2 Combinations of parameters at time of RRT and hospital mortality**

| **Parameter at time of RRT** | **Serum pH <7.2** | **Urine**  **<400ml/24hrs** | **SOFA score >12** | **MAP**  **≤ 65** | **CVS failure** | **RESP failure** | **HEP failure** |
| --- | --- | --- | --- | --- | --- | --- | --- |
| **Serum pH <7.2** | 79.5% |  |  |  |  |  |  |
| **Urine <400ml/24hrs** | 83.8% | 68.9% |
| **SOFA score >12** | 87.5% | 76.5% | 72.2% |
| **MAP ≤ 65** | 82.9% | 77.8% | 78.7% | 71.7% |
| **CVS failure** | 79.7% | 76.1% | 77.9% | 75.4% | 70.2% |
| **RESP failure** | 84.9% | 77.2% | 78.4% | 77.7% | 76.4% | 69.5% |
| **HEP failure** | 92% | 83.6% | 82.4% | 86.7% | 92.3% | 87.5% | 80.8 |
| **Total OF 1 ***  **Total OF 2 ***  **Total OF 3 ***  **Total OF >3 *** | 69.4%  75.7%  80.8%  92% | 53.3%  65.5%  77%  91.2% | 59%  64.9%  75%  91.1% | 62.9%  68.2%  77.5%  90% | - | - | - |
| **Days since ICU admission**  **<3**  **3 - 5**  **6 – 10**  **>10** | 77.3%  94.7%  91.7%  84.6% | 66.9%  76.5%  70.4%  87.9% | 70.8%  75.2%  71.6%  85.7% | 69.6%  77.4%  74.2%  85.4% | 69.9%  74.1%  65.9%  76.9% | 68.8%  70.1%  65.6%  80.8% | 79.5%  76.9%  87.5% 100% |

*Abbreviations: ICU= intensive care unit; OF = failed organ system(s); SOFA = Sequential Organ Failure Assessment; CVS = cardiovascular; RESP = respiratory; MAP = mean arterial blood pressure in mmHg; HEP = liver*

** excluding AKI*

**Table S3 Multivariate analysis: Parameters on day of RRT affecting hospital outcome of patients with AKI treated with RRT**

(Exclusion of patients with missing data for creatinine, urea, MAP, ph;

patients included n= 1,794)

| **Parameter** | **B** | **S.E.** | **p** | **OR** | **95.0% C.I.** |
| --- | --- | --- | --- | --- | --- |
| **pH on day of RRT** | -3.692 | .457 | 0.0001 | 0.025 | 0.01 – 0.06 |
| **[creatinine] on day of RRT** | -0.002 | 0.00 | 0.0001 | 0.998 | 0.997 – 0.999 |
| **[urea] on day of RRT** | .004 | .001 | 0.0001 | 1.004 | 1.002 – 1.006 |
| **age** | .034 | .004 | 0.0001 | 1.035 | 1.03 – 1.04 |
| **RESP failure on day of RRT** | .418 | .116 | 0.0001 | 1.52 | 1.21 – 1.91 |
| **GI failure on day of RRT** | .628 | .139 | 0.0001 | 1.87 | 1.43 – 2.46 |
| **Oligoanuria on day of RRT** | .647 | .115 | 0.0001 | 1.91 | 1.52 – 2.39 |
| **Pre-existing chronic illness** | .664 | .133 | 0.0001 | 1.94 | 1.50 – 2.52 |
| **HAEM failure on day of RRT** | .809 | .219 | 0.0001 | 2.25 | 1.46 – 3.45 |
| **HEP failure on day of RRT** | .844 | .282 | 0.0001 | 2.33 | 1.34 – 4.04 |
| **ventilated** | .878 | .210 | 0.0001 | 2.41 | 1.59 – 3.63 |
| **NEURO failure on day of RRT** | .896 | .206 | 0.0001 | 2.45 | 1.64 – 3.67 |
|  |  |  |  |  |  |
| **CVS failure on day of RRT** | .074 | .129 | 0.566 | 1.08 | 0.84 – 1.39 |
| **male gender** | .065 | .121 | 0.59 | 1.07 | 0.84 – 1.35 |
| **post non-surgical admission** | .196 | .195 | 0.31 | 1.217 | 0.83 – 1.78 |
| **post cardiac surgery** | -.239 | .213 | 0.261 | 0.79 | 0.52 – 1.195 |
| **post - emergency surgery** | .286 | .216 | 0.185 | 1.332 | 0.87 – 2.03 |
| **Constant** | 26.568 | 3.343 | 0.0001 | 345540770279.732 |  |

*Abbreviations: RRT = renal replacement therapy; CVS = cardiovascular; RESP = respiratory; HAEM = haematological; GI = gastrointestinal; NEURO = neurological; HEP = hepatic*

The area under the ROC curve was 0.78
